# Hyphenating size‐exclusion chromatography with electrospray mass spectrometry; using on‐line liquid‐liquid extraction to study the lipid composition of lipoprotein particles

**DOI:** 10.1002/rcm.7301

**Published:** 2015-10-05

**Authors:** Michael Osei, Julian L. Griffin, Albert Koulman

**Affiliations:** ^1^ Medical Research Council Human Nutrition ResearchElsie Widdowson Laboratory120 Fulbourn RoadCambridgeCB1 9NLUK

## Abstract

**Rationale:**

Lipoproteins belong to the most commonly measured clinical biochemical parameters. Lipidomics is an orthogonal approach and aims to profile the individual lipid molecules that jointly form the lipoprotein particles. However, in the first step of the extraction of lipid molecules from serum, an organic solvent is used leading to dissociation of the lipoproteins. Thus far it has been impossible to combine lipidomics and lipoprotein analysis in one analytical system.

**Methods:**

Human plasma was diluted in phosphate‐buffered saline (PBS) and injected onto a Superose 6 PC 3.2 column with PBS as a mobile phase to separate lipoproteins. The eluent was led to a Syrris FLLEX module, which also received CHCl_3_/MeOH (3:1). The two phases were mixed and subsequently separated using a Teflon membrane in an especially designed pressurized flow chamber. The organic phase was led to a standard electrospray source of an Orbitrap mass spectrometer.

**Results:**

Size‐exclusion chromatography (SEC) has been commonly applied to separate lipoproteins and is considered a practical alternative to ultracentrifugation. Through the on‐line liquid‐liquid extraction method it becomes possible to obtained detailed mass spectra of lipids across different lipoprotein fractions. The extracted ion chromatograms of specific lipid signals showed their distribution against the size of lipoprotein particles.

**Conclusions:**

The application of on‐line liquid‐liquid extraction allows for the continuous electrospray‐based mass spectral analysis of SEC eluent, providing the detailed lipid composition of lipoprotein particles separated by size. This approach provides new possibilities for the study of the biochemistry of lipoproteins. © 2015 The Authors. Rapid Communications in Mass Spectrometry Published by John Wiley & Sons Ltd.

Most lipids, being poorly soluble in water, are transported through the circulation as complexes with proteins, called lipoprotein particles. These are diverse in size and composition; based on the hydrated density divided into the following fractions: HDL (High Density lipoprotein), LDL (Low Density lipoprotein) and VLDL (Very Low Density lipoprotein) also called "triglyceride‐rich lipoprotein" fraction.^1^ It has been shown that the original separation into three classes is too limited and that there are more intermediate classes of lipoproteins. Different densities and sizes are obtained by the combination of different proteins that maintain the organisation of lipoprotein particles. The actual lipid composition of each of the fractions is becoming more relevant now lipidomic techniques are able to give a detailed profile of the lipids in a blood sample. This detailed information on the levels of specific lipids in blood plasma raises questions about the organisation of these lipids and if these are specific for certain lipoprotein particles or fractions.

Currently, there are a number of different technologies that can separate or detect the different classes of lipoproteins. The gold standard for the separation of lipoproteins is ultra‐centrifugation, which separates the lipoproteins by density. However, the density cut‐off between fractions cannot be objectively defined and the method is very time‐consuming. Therefore, other separation methods have been developed. Methods have been published based on differential precipitation^2^ or electrophoresis.^3^ For over 30 years people have been working on chromatographic separation of lipoproteins.^4^ The chromatographic separation of lipoproteins is possible based on size exclusion. Size‐exclusion chromatography (SEC), also known as gel filtration chromatography or gel permeation chromatography, is able to retain the smaller particles or molecules due to their ability to diffuse into the pores of the column material which causes their retention while larger particles or molecules that are too big to diffuse into the pores will be less retained. Since the start of lipoprotein separation by SEC two column materials have been used, TSK G5000^4^ and later on Superose 6.^5^ These two column materials are still the materials of choice 20 years later, only the column dimensions have changed.^6^ Over the past 30 years the main developments have been on the detection methods of the cholesterol and other lipids in the eluents. Different groups have developed post‐column reactions with either enzymes or reagents to detect cholesterol, cholesteryl esters, triglycerides or other lipid classes.^4^, ^5^, ^7^, ^8^, ^9^, ^10^, ^11^ It is also possible to collect fractions from SEC‐based separations for subsequent analysis.^12^ Recently, collected fractions have been used in proteomics^13^ and lipidomics approaches using either electrospray ionization (ESI)^14^ or matrix‐assisted laser desorption/ionization (MALDI).^15^

The combination with mass spectrometry opens the possibility to determine both the different size and abundance of lipoproteins as well as the lipid composition of the lipoprotein particles. Different groups have used ESI‐based mass spectrometry (MS) to determine the lipid composition of the fraction collected by either ultra‐centrifugation^16^, ^17^ or SEC.^14^, ^18^ Thus far it has not been possible to directly hyphenate SEC with ESI‐based MS. The main problem is that optimal separation with all SEC column materials is dependent on non‐volatile buffers. The salt concentrations of these buffers make it practically impossible to maintain a stable spray into the MS using ESI, giving corona discharge in the ESI source and rapidly blocking the orifice of the MS through the build‐up of salts. Many people have tried to develop a sustainable hyphenation between SEC and ESI‐MS, but thus far the only workable solution has been to switch to volatile buffers.^19^ However, these approaches do not work with lipoproteins and in many cases not with other types of SEC‐based separations, which are dependent on specific salt concentrations in the buffer.

Our aim was to develop a method to hyphenate ESI‐MS directly with SEC, which would allow us to determine directly the lipid composition of lipoprotein particles.

## Experimental

### Reagents

All solvents (all HPLC grade), and all other chemicals were purchased from Sigma (Darmstadt, Germany).

### Characteristics of blood donors

Blood plasma was obtained from five healthy blood donors and samples were collected at three different time points. Donors did not take any medication within 2 weeks before blood drawing. We received informed consent of all donors in written form. All blood samples were collected in EDTA monovettes (Sarstedt, Leicester, UK) by venipuncture performed by trained phlebotomists. The blood samples were immediately placed on ice after collection and then centrifuged for 15 min at 1962 *g* at 4°C. The plasma was collected in new tubes. Participants were provided with an energy‐balanced lunch at 12:00 on the first study day. The first blood sample was taken at 12:45. An energy‐balanced dinner was provided at 17:00 and a second (fasting) blood sample was taken at 08:30, followed by breakfast (09:00) and a third blood collection at 10:30. All meals were energy balanced with a macronutrient energy composition of 50% carbohydrate, 35% fat, and 15% protein.

### Equipment

For fast protein liquid chromatography (FPLC), a Shimadzu Prominence UFLC system with three LC‐20 AD pumps and SPD‐M20A photodiode array detector was used. The mass spectrometry system consisted of an LTQ Orbitrap Velos (Thermo, Bremen, Germany) with a standard ion max ESI source (voltage: 4.0 kV; capillary temp: 275°C; sheath gas: 20 arbitrary (arb) units; Aux gas: 10 arb; Sweep gas: 5 arb). Generally, the selected masses were isolated with a 1.5 *m/z* width in the linear ion trap and then fragmented using either the linear ion trap with 35% relative collision energy or in the HCD (Higher‐energy Collision‐induced Dissociation) collision cell, with a range of collision energies from 5% to 75% relative collision energy. All spectra were recorded in the Orbitrap set at 100,000 resolution.

### Lipoprotein separation by FPLC

Freshly thawed blood plasma samples were diluted 1:5 with phosphate‐buffered saline (PBS) before separation using a full‐loop injection of 5 μL. Lipoprotein particles were separated using the previously described method^18^ using a Superose 6 PC 3.2/30 column (GE Healthcare Europe GmbH, Munich, Germany) with PBS as the mobile phase (at 50 μL min^–1^).

### On‐line liquid‐liquid extraction

**Figure 1:**
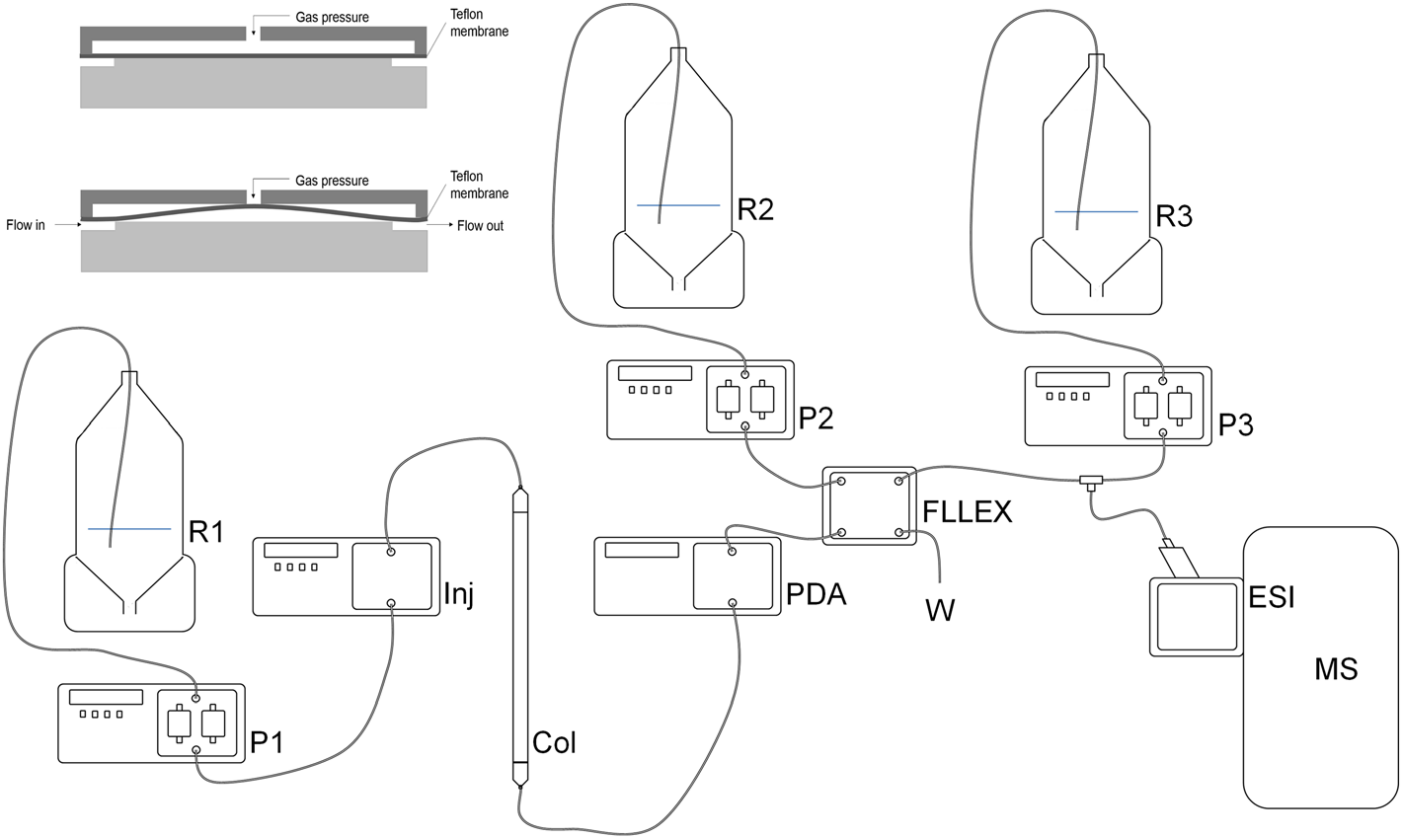
Schematic of the instrumental setup. R1: buffer for separation on column; P1: column pump; Inj: autosampler; Col: column; PDA: photodiode‐array detector; FLLEX, on‐line liquid‐liquid extraction unit; R2: reservoir containing organic solvent for liquid‐liquid extraction; P2: organic phase pump; R3: reservoir containing ionization modifier. P3: pump; ESI: electrospray source; MS: mass spectrometer; W: waste. In the top left there is a schematic of the on‐line liquid‐liquid flow cell.

A schematic overview of the instrumental set‐up is shown in Fig. 1. The eluent was led to an Asia FLLEX module (Syrris Ltd, Royston, UK), which also received CHCl_3_/MeOH (3:1) at 50 μL min^–1^. Using a 250 μL loop the two phases were mixed and subsequently separated using a Teflon membrane in the especially designed pressurized glass flow chamber of the Asia FLLEX module. This yielded both the aqueous phase and the organic phase as separated eluents. The organic phase was mixed with a flow of isopropanol/methanol 7.5 mM NH_4_Ac at 150 μL min^–1^ and led to a standard ESI source. Spectra were obtained using the LTQ Orbitrap Velos.

## Results

While lipoprotein particles should theoretically be able to ionise in electrospray, their size is at the upper limit of protein complexes that have been successfully brought into the gas phase,^20^ and only the smallest HDL particles have been successfully ionized.^21^ The break‐up of these particles in the gas phase would also be theoretically possible but the trapping of the individual lipid ions associated with these complexes would be extremely challenging. This meant that for the analysis of the individual lipids of the lipoprotein particle we would have to rely on some sort of in‐solution dissociation of the lipoprotein particles after the separation by SEC.

In classic lipid analysis, for instance, using the Folch extraction,^22^ lipids are extracted by liquid‐liquid partitioning, where a mixture of chloroform/methanol dissociates the lipoprotein particles and the lipids are taken up by the organic phase. To be able to measure the individual lipids by ESI‐MS it is essential to dissociate these from the lipoproteins. Therefore, our initial work was focused on dissociating the lipids from the lipoproteins post‐column. Different attempts were made through the post‐column addition of organic solvents (like, CHCl_3_, CHCl_3_/MeOH, and MeOH/IPA, etc.) at much higher flow rates (200 to 500 μL min^–1^) than used for the chromatography, with the aim to dissociate the lipoprotein particles. Our aim of the post‐column addition of these organic solvents was to achieve a similar dissociation as in classical lipid extraction but maintain the versatility and convenience of an on‐line method. To achieve such a method we used adjustable flow splitters, with the aim to have the eluent with a highly reduced salt concentration arriving at the ESI probe. However, we found that the levels of salt were still too high to maintain a stable spray. Further dilution through higher flow rates of the post‐column addition were not practical and compromised the sensitivity of the lipid analysis. This suggested that we needed to find a system that would not only be able to dissociate the lipoprotein particles, but also help to separate the organic phase from the salt‐containing buffer.

A solution was found from the flow chemistry literature.^23^ The field of flow chemistry uses a continuous flow in which chemical synthesis is performed, for instance the mixing of a flow of reagents with a flow of a catalyst through a coil at a specific temperature to initiate the reaction. The product of such a reaction usually needs some type of purification, and on‐line liquid‐liquid partitioning would be suitable. Figure 1 shows the schematic setup for the lipoprotein separation and analysis with a Hart system from Syrris (Royston, Hertfordshire, UK) that allows on‐line liquid‐liquid partitioning using microfluidic setup. The system, named FLLEX, is based on two glass slides that when combined form a microfluidic channel through which solvents can flow. Between the glass slides is a Teflon membrane with pores of 0.22 µm diameter (see Fig. 1). The hydrophobic nature of the Teflon repels the aqueous phase, but the organic phase passes through the pores. The mixed phases flow through the channel and at the end of the channel there are outlets in both glass slides. To stabilise the partitioning, the flow is held at a constant back pressure, using nitrogen, with a small pressure off‐set (ca 200 mbar) that can be adjusted to obtain separation of the two layers.

This allowed us to develop a new method that gives information about both the size of the lipoprotein fractions and their detailed lipid composition in one analysis of a relatively small volume of plasma or serum (in this case 1 μL) using a novel combination of flow chemistry, analytical chemistry and biochemical analysis. Although every step of the method has been applied previously in one or more fields, this report is the first to show that a successful combination of these three fields can yield technology that gives insight into the composition of lipoproteins and their size.

The separation of the lipoproteins was achieved at a flow rate of 50 μL min^–1^ of the PBS. Post‐column the eluents were led through a PDA detector and subsequently connected to the FLLEX system. Through a second pump an organic solvent (mixture of CHCl_3_/MeOH 2:1) was delivered to the second connector of the FLLEX at the same flow rate of 50 μL min^–1^. The mixture of the two solvent phases was led through a 250 μL loop before entering the microfluidic channel. The backpressure was set at 4 bar with a differential pressure of 0.2 bar, which yielded a stable separation between the two phases. The aqueous phase was led to waste. A third pump was used to deliver a mixture of IPA/MeOH containing 7.5 mM NH_4_Ac, to facilitate a stable spray in the ESI source and provide NH_4_^+^ for the ionisation of neutral lipids.^24^

**Figure 2:**
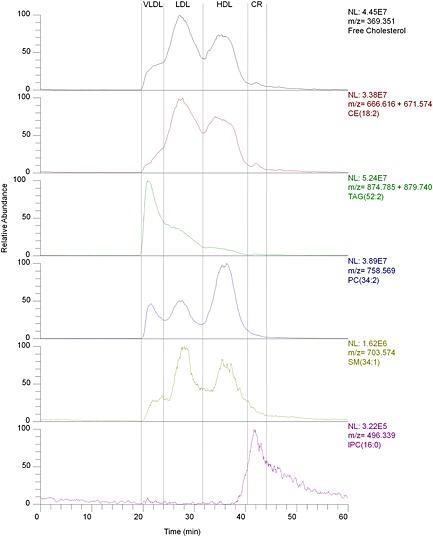
Extracted ion chromatograms of five lipid classes. Top line (black) free cholesterol, second line (red) cholesteryl ester [CE(18:2)+NH_4_]^+^ and [CE(18:2)+Na]^+^; line 3 (green): triglycerides [TG(52:3)+NH_4_]^+^ and [TG(52:3)Na]^+^; line 4 (blue) phosphocholine [PC(34:2)+H]^+^, line 5 (yellow) sphingolipid [SM(34:1)+H]^+^; line 6 (purple) lysophosphocholine [lysoPC(16:0)+H]^+^.

The separation of the lipoproteins from blood plasma could be readily achieved as previously documented (Fig. 2) with very similar retention times and with the same limitation. The Superose 6 PC column is not able to separate IDL from VLDL particles (which is described here as VLDL).The resolution of this separation was limited and there was considerable overlap between the different lipoprotein fractions, which has also been reported by other groups. The on‐line liquid‐liquid extraction introduced additional peak broadening, but this could be limited by using a relatively small volume (250 μL) for the mixing loop in the FLLEX system and small diameter tubing. The delay of the peaks was around 4–5 min between the PDA detector and the mass analyser.

The method was stable and reproducible, although protein denaturation could cause the membrane to block, which happened after 20 to 30 samples. This blockage of the membrane resulted in impaired separation of the two phases and a loss of signal. Replacement of the membrane solved this without any further problems and did not demand any changes to the setting or alteration of the system and would not require more than 5 min.

The technology has two major advantages over what has been published in the field of lipoprotein analysis based on size exclusion. The method is faster than any of the previous approaches that rely on mass spectrometry. All previous work collected fractions and had to rely on off‐line sample work‐up to determine the lipid composition of these fractions. This is very time consuming and labor intensive The second main advantage of our method is that through the combination with high‐resolution mass spectrometry (HRMS) it is possible to determine the contribution of specific lipids to the different lipoprotein fractions. Furthermore, HRMS allows the extraction of ions with very small mass windows. This means that only the intensity of ions with that particular molecular formula contribute to the signal.^25^ With careful analysis of the retention time it becomes possible to determine if specific lipids are dependent of the size of the lipoprotein or even determine the size of lipoproteins.

**Figure 3:**
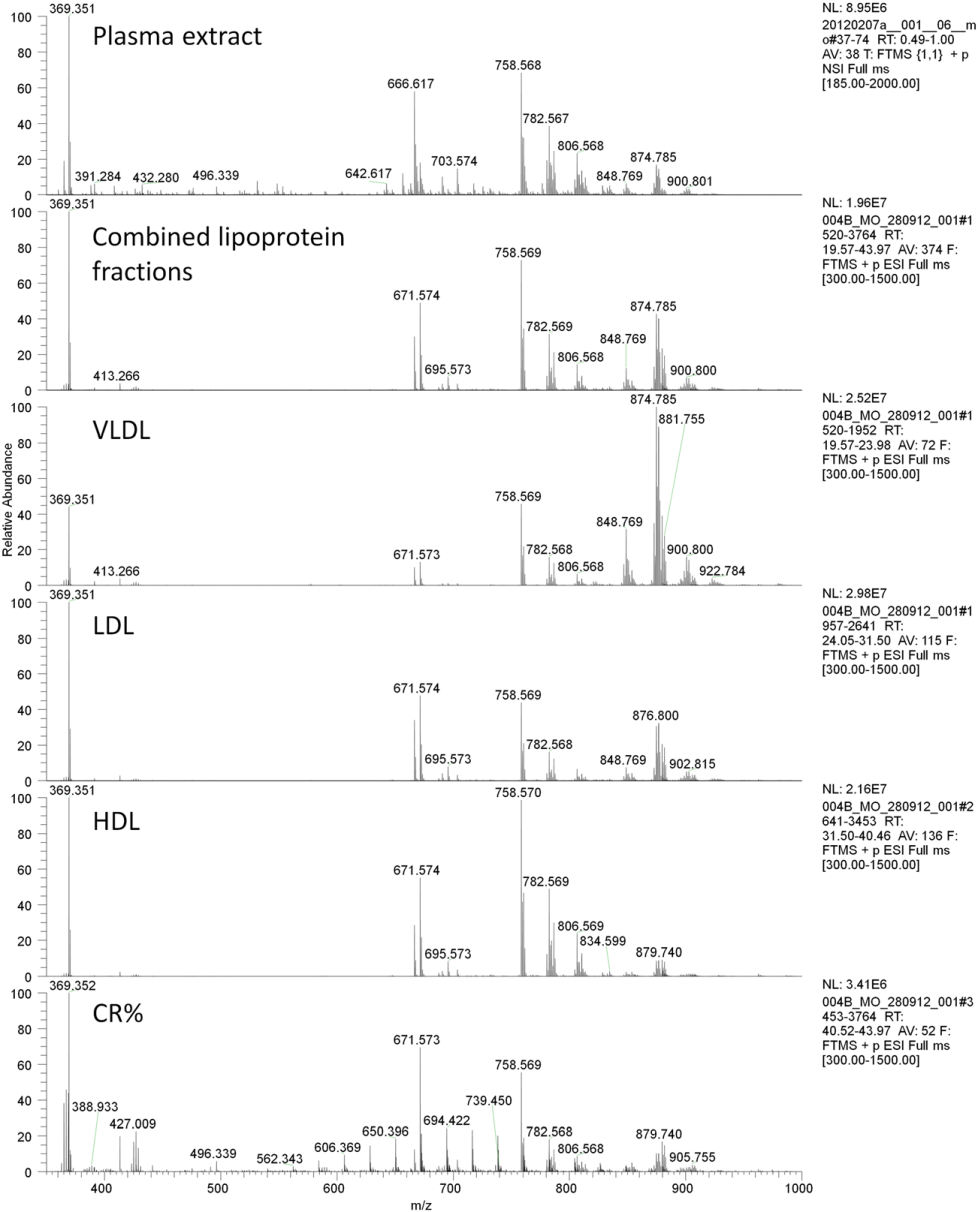
Average mass spectrum (350–1200 *m/z*) of the different lipoprotein fractions in comparison to a lipid extract of the same plasma sample before separation of the lipoproteins. Plasma is from a healthy human after 12 h of fasting. Although most lipids from the total lipid extract can be found in one or more of the lipoprotein fractions there are also a number of lipids for which the levels are much lower in any of the lipoprotein fractions than in the complete extract, showing that not all lipids are circulating as part of lipoprotein particles.

The SEC separation of plasma using a Superose 6 column led to three main peaks (see Fig. 2). These were the three main fractions of lipoproteins and showed clear differences in their lipid composition (see Fig. 3). The VLDL fraction mainly consisted of triglycerides and cholesterol/cholesteryl esters and eluted between 19 and 24 min. The LDL fraction consisted mainly of cholesterol (esterified and free) and eluted between 24 and 32 min. The HDL fraction had a relatively large amount of phospholipids and eluted between 31 and 41 min. A fourth fraction, which eluted between 40 and 49 min, and at a much lower level than the previous three, was the chylomicron remnant. It contained less phospholipids and more triglycerides than the HDL fraction, it also contained more lyso‐phospholipids.

For the identification of the lipid species we relied on the high resolution and mass accuracy of the Orbitrap mass analyser. HRMS enabled the separation of lipids to a molecular formula level, but not beyond (it cannot separate isobaric species). The resolution of 100,000 at 400 *m/z* as used in our setup allowed for the baseline separation of, for instance, molecular formulae C_41_H_78_NPO_8_+H^+^ (*m/z* 744.554) and C_42_H_82_NPO_7_+H^+^ (*m/z* 744.590), but was unable to determine if C_41_H_78_NPO_8_+H^+^ is PC(33:2) or PE(36:2). The inability to separate isobaric lipids also means that we could not identify the individual fatty acids within an intact lipid. Therefore, we used a nomenclature that does reflect this ambiguity, so the ion 900.801 *m/z* (C_57_H_102_O_6_+NH_4_^+^) was identified as TG(54:4), which meant that we identified it as a triacylglycerol with 56 carbon atoms and 4 double bonds across the three fatty acids, but we could not asses if this was either TG(18:0;16:0;20:4), TG(18:2;18:2;18:0) or any other fatty acid combination that would have led to this number of carbons and double bonds.

The ability to determine in detail the lipid composition of the lipoprotein fractions separated by size revealed some novel insights. For LDL and HDL the ratio between free cholesterol (measured as 369.351 *m/z*; [CE–H_2_O+H]^+^) and cholesteryl linoleate (measured as the ions 666.616 *m/z* [CE(18:2)+NH_4_]^+^ and 671.573 *m/z* [CE(18:2)+Na]^+^) was the same, while for the large VLDL particles the ratio was different, with an increased amount of free cholesterol (see Fig. 3). The largest VLDL particles contained relatively more free cholesterol. The other striking observation was that in all of the lipoprotein particles the cholesteryl esters were present as cholesteryl linoleate (CE(18:2)). In most samples between 85% and 90% of all was CE(18:2), while plasma cholesteryl esters usually contain around 52% linoleate.^26^ The levels of cholesteryl palmitate (CE(16:0)), cholesteryl oleate (CE(18:1)) and cholesteryl arichidonate (CE(20:4)) were much lower than in lipid extracts from plasma. Both methods rely on liquid/liquid extractions and it is fair to assume that these lipids have the same affinity for lipoprotein particles, which suggests that these cholesteryl esters are not only part of the lipoproteins, but are also trafficked differently through the circulation.

**Figure 4:**
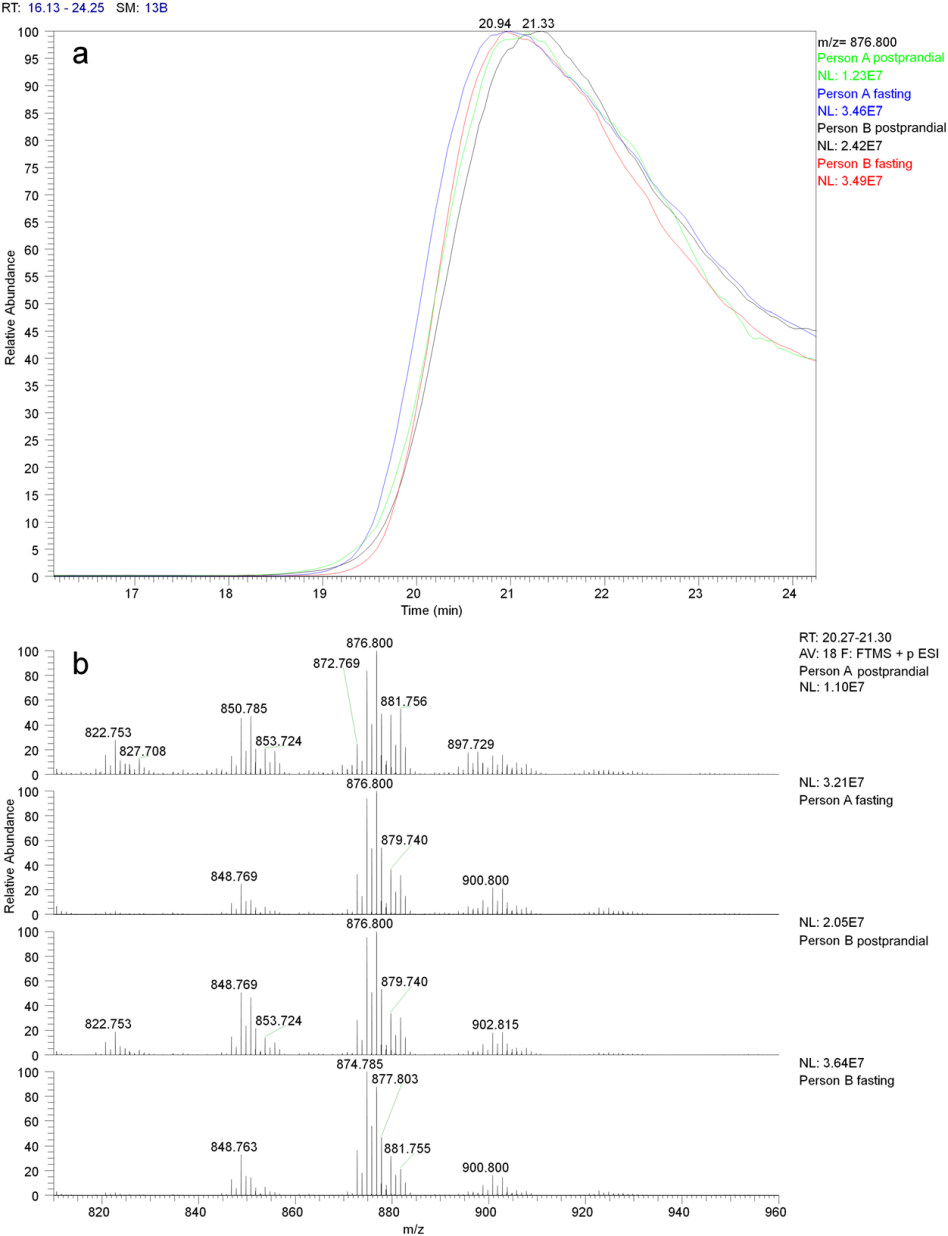
(a) The difference in the VLDL lipoproteins in size and composition in fasting and non‐fasting state. Fasting leads to an increase in the VLDL particle size (lower retention time). (b) The average mass spectra (from Rt 20.3–21.3 min) of the VLDL fractions of fasting and non‐fasting state. Fasting leads to a decrease in triglycerides with shorter more saturated fatty acids such 822.753 *m/z* TG(48:1).

With this approach we were able to show that the size of the VLDL particles increased. We based this on the intensity of the ion 786.800 *m/z* (TG(52:2)) which reached its maximum earlier in fasting samples compared to post‐prandial samples from the same person (see Fig. 4). As SEC retains smaller particles better than larger particles it could be concluded that fasting leads to VLDL size increase. In addition we could compare the triglyceride composition which showed that the fasting VLDL contained relatively longer chain unsaturated fatty acids. This was most notable for the ions from TG(48:1) which were clearly present in the VLDL particles in the post‐prandial samples, while in fasting samples the level was much lower. The increase in desaturation could also be detected in the ratio between ions 900.800 and 902.815 (TG(54:4) and TG(54:3)). In the fasting samples the 900.800 ion was more abundant than that at 902.815 and in the post‐prandial samples this was the other way around.

The small TGs, like TG(48:1), which were present in the VLDL fraction during the non‐fasting state, have recently been implicated as markers of increased relative risk for type 2 diabetes.^27^ From our own work it appears that these small TGs contain fatty acids such as palmitate (C16:0) and myristate (C14:0) that are products of *de novo* lipogenesis. In these samples we could show that these palmitate‐ and myristate‐containing TGs are more common for the non‐fasting status and mainly occur in the VLDL fraction, while other TGs that contain very long‐chain fatty acids increase during fasting and in non‐fasting. These larger TGs are less specific for VLDL particles.

There is further room to improve the method. The column material used in this study for SEC was not able to retain the chylomicrons and therefore this fraction was not captured. The use of different column materials^8^ or a combination of columns might improve the capture of lipoprotein particle sizes. A second improvement would be the detection of the specific proteins that form the lipoprotein particles; this would enable us to give a more specific classification of the lipoproteins and not only by size. The method does not have to be based on lipoproteins and opens the way to a generic hyphenation of SEC with ESI‐MS.

## Conclusions

The direct hyphenation between size‐exclusion chromatography and electrospray ionisation mass spectrometry through on‐line liquid‐liquid extraction is a new tool in the study of lipid metabolism. It is a more simple method than any current methodologies for lipoprotein separation and does not rely on fractionation for further analysis. For the first time it gives the opportunity to determine the contribution of specific lipids to lipoprotein particles of different sizes.
